# A novel treatment approach to the novel coronavirus: an argument for the use of therapeutic plasma exchange for fulminant COVID-19

**DOI:** 10.1186/s13054-020-2836-4

**Published:** 2020-04-02

**Authors:** Philip Keith, Matthew Day, Linda Perkins, Lou Moyer, Kristi Hewitt, Adam Wells

**Affiliations:** 1 Critical Care Medicine, Lexington Medical Center, 2720 Sunset Boulevard, West Columbia, SC 29169 USA; 2 Critical Care Medicine, Novant Health Forsyth Medical Center, 3333 Silas Creek Parkway, Winston Salem, NC 27103 USA

The novel coronavirus (“SARS-CoV-2”) outbreak has created a sense of panic globally and has the medical community rapidly searching for answers. An estimated 100,000 individuals have already been infected with nearly 3300 deaths attributed to the disease (termed COVID-19) [1]. The search for effective treatment is underway with multiple investigations ongoing across the world. Chinese authorities have reported success treating infected patients with donated plasma from survivors of the illness, the proposed benefit being protective antibodies formed by the survivors [2]. Plasma transfusion and blood purification are not novel therapies, and we propose therapeutic plasma exchange as a possible treatment for fulminant COVID-19.

With COVID-19, the degree of illness varies, ranging from asymptomatic to fulminant and fatal. The World Health Organization estimates that serious illness may occur in as many as 13.8% of cases and 6.1% are critical [3]. When fulminant, patients may develop sepsis, acute respiratory distress syndrome (ARDS), and/or multiple organ failure which are not unique to coronavirus. While treatment of the virus itself is certainly desired, treatment of the systemic response is likely to be the more important aspect of care and should be aggressively sought. This host response to infection has been well described and involves a complex interaction of cytokine storm, inflammation, endothelial dysfunction, and pathologic coagulation [4–8]. The pathway is common to multiple inciting events and has been the target of treatment for years, with therapeutic plasma exchange uniquely offering benefit on multiple levels by removing inflammatory cytokines, stabilizing endothelial membranes, and resetting the hypercoagulable state [4, 8, 9]. An in-depth review is beyond the scope of this editorial, but the reader is encouraged to review the referenced articles. Figure 1 briefly illustrates the pathway.

**Fig. 1:**
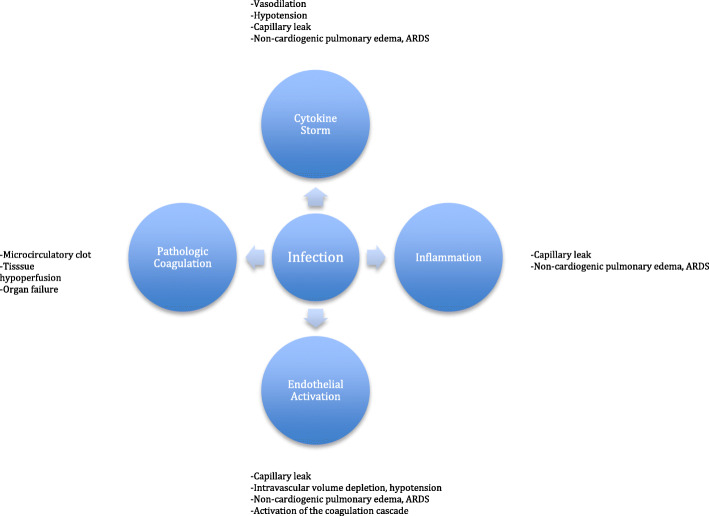
Physiologic pathway of sepsis which serve as potential targets of therapeutic plasma exchange

Busund and colleagues showed a tendency toward improved mortality with adjunct TPE in adult patients with sepsis and multiple organ failure in the sole, adult-only randomized controlled trial on this subject [10] while a meta-analysis by Rimmer showed mortality benefit in adult patients as well [11]. Drawing from this data, Patel and colleagues utilized TPE during the 2009 H1N1 influenza A outbreak in three pediatric patients presenting in a similar fashion to those seen with fulminant COVID-19 today [12]. All three patients developed ARDS with hemodynamic compromise that continued to deteriorate despite standard care and rescue therapy for ARDS including inhaled nitric oxide (3/3) and veno-venous ECMO (1/3). Predicted mortality was high, but all three had full recovery from their illness after receiving rescue TPE.

Others have reported successful outcomes, feasibility, and safety of TPE for sepsis, but none have investigated specifically in pneumonia/ARDS. Our group has recently submitted the results of our single-center experience with TPE in sepsis with multiple organ failure with the manuscript currently under consideration for publication with preprint available online (DOI: 10.21203/rs.3.rs-16022/v1) [13]. In our trial, charts were retrospectively reviewed and patients receiving adjunct TPE were propensity matched to patients with similar illness who received standard of care alone. Full details are available online, but it should be noted that all patients required ≥ 2 vasopressors and all patients receiving TPE required mechanical ventilator support. Nearly half of the patients in both groups (39/80) presented with pneumonia as the primary source of infection, and a subgroup analysis showed the greatest mortality benefit with TPE in these patients (47.8% mortality vs. 81.3% mortality, *p* = 0.05). While a single-center, retrospective trial is obviously limited, the results are very encouraging and support the need for further investigation, particularly in today’s environment with increasing incidence of COVID-19. Our practice has changed based on our experience, and we now often utilize TPE earlier in the clinical course of septic shock with MODS and ARDS rather than as “rescue therapy.” Anecdotally, the results have been remarkable but have not been reviewed or statistically analyzed.

The novel coronavirus has generated worldwide attention due to the potential impact on global health. The uncertainties of the disease are frightening, but it is likely that the host response to coronavirus is the same as that seen in other infections. Presently, treatment for sepsis and ARDS centers on early antimicrobials, source control, and “supportive care.” This outbreak should serve as impetus to investigate therapies targeting the pathways that lead to the morbidity and mortality associated with these syndromes. Therapeutic plasma exchange shows promise, and we propose that randomized trials be designed to investigate further.

## Data Availability

Not applicable.
